# The International Medical Congress

**Published:** 1885-03

**Authors:** 


THE INTERNATIONAL MEDICAL CONGRESS.—The organization of the Section of Dental and Oral Surgery of this Medical Congress, which meets in Washington City in 1887. Ninth Session, and of which Professor John S. Billings, M. D., of the Johns Hopkins University and the U. S. Army, is Secretary General, has been completed by the appointment of the following gentlemen:

*President.*—Jonathan Taft, M. D., D. D. S., Cincinnati, Ohio;

*Vice President.*—W. W. Allport, M. D., D. D. S., Chicago, Ill; William H. Dwinelle, M. D., D. D. S., New York City; J. L. Williams, M. D,, D. D. S., Boston Mass.

*Secretaries.*—E. A. Bogue, M. D , D. D. S., New York City; Geo. H. Cushing, D. D. S., Chicago, Ill.

*Members of Council.*—W. C. Barrett, M. D., D. D. S., Buffalo, N. Y; Thos. Fillebrown, M. D., D. M. D., Portland, Me; F. J. S. Gorgas, M. D., D. D. S., Baltimore, Md; Edw. Maynard, M. D., Washington, D. C; J. H. McKellops, D. D. S., St. Louis, Mo. W. H. Morgan, M. D., D. D. S., Nashville, Tenn; C. N. Pierce, D. D. S., Philadelphia, Pa; L. D. Shepard, D. D. S., Boston, Mass; Jas. Truman, D. D. S., Philadelphia, Pa. James W. White, M. D., D. D. S., Philadelphia, Pa.

